# Effective Band Structure and Crack Formation Analysis in Pseudomorphic Epitaxial Growth of (In_*x*_Ga_1–*x*_)_2_O_3_ Alloys: A First-Principles Study

**DOI:** 10.1021/acsomega.3c10047

**Published:** 2024-03-20

**Authors:** Mohamed Abdelilah Fadla, Myrta Grüning, Lorenzo Stella

**Affiliations:** † School of Mathematics and Physics, Queen’s University Belfast, University Road, Belfast BT7 1NN, U.K.; ‡ School of Chemistry and Chemical Engineering, Queen’s University Belfast, Stranmillis Road, Belfast BT9 5AG, U.K.; § European Theoretical Spectroscopy Facility (ETSF)

## Abstract

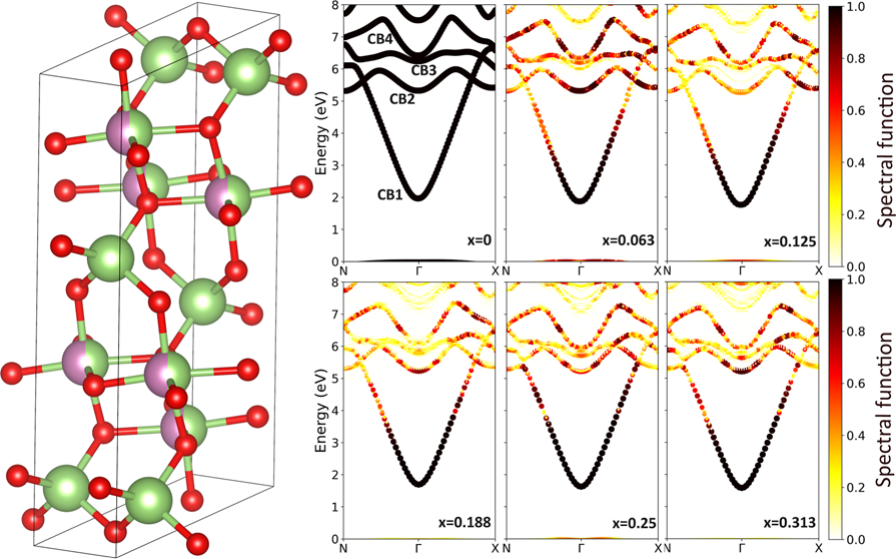

Ga_2_O_3_ is a promising material for power electronic applications. Alloying with In_2_O_3_ is used for band gap adjustment and reduction of the lattice mismatch. In this study, we calculate the effective band structure of 160-atom (In_*x*_Ga_1–*x*_)_2_O_3_ supercells generated using special quasi-random structures where indium atoms preferentially substitute octahedral gallium sites in β-Ga_2_O_3_. We find that the disorder has a minimal effect on the lower conduction bands and does not introduce defect states. Employing the Heyd, Scuseria, and Ernzerhof (HSE06) hybrid functional, we accurately model the band gap, which remains indirect for all considered indium fractions, *x*, linearly decreasing from 4.8 to 4.24 eV in the range of *x* ∈ [0, 0.31]. Accordingly, the electron effective mass also decreases slightly and linearly. We determined the critical thickness for epitaxial growth of the  alloys over β-Ga_2_O3 surfaces along the [100], [010], and [001] directions. Our findings offer new insights into site preference, effective band structure, and crack formation within  alloys.

## Introduction

Wide band gap semiconductor (WBGS) oxides have captured much attention as emerging materials for power electronic devices because of their improved properties compared to conventional materials such as GaN and SiC.^1,2^ Potential applications of WBGS to devices include metal oxide–semiconductor field-effect transistors and Schottky barrier diodes (SBDs).^3,4^ One of the most actively studied WBGS is β-Ga_2_O_3_, which has several desirable characteristics: with a band gap of approximately 4.8 eV, it is transparent in the UV–visible range; it is the most stable Ga_2_O_3_ polymorph, with a melting temperature of 1800 °C;^5,6^ a low production cost in the form of pure and high-quality substrate;^7,8^ and a predicted breakdown electric field of 8 MV/cm,^3^ which is larger than traditional materials.

β-Ga_2_O_3_ crystallizes in the base-centered monoclinic space group 12 (*C*2/*m*), with 20 atoms per unit cell.^9^ Inequivalent gallium atoms occupy two distinct positions, denoted Ga_I_ and Ga_II_, and form tetrahedra or octahedra, respectively, with three inequivalent oxygen atoms (O_I_ and O_II_, threefold) and (O_III_, fourfold). Fourfold coordinated O_III_ connects three octahedral and one tetrahedral gallium sites. Among the threefold coordinated oxygen atoms, O_I_ connects two octahedral and one tetrahedral gallium sites, while O_II_ connects two tetrahedral and one octahedral gallium sites. Low symmetry leads to highly anisotropic properties and offers a wide range of growth possibilities. Like other oxides, β-Ga_2_O_3_ tends to exhibit unintentional n-type conduction,^10,11^ while achieving p-type is challenging.^12,13^

 alloying allows one to adjust and tune β-Ga_2_O_3_ intrinsic properties, including band gap engineering and strain by lattice misfit reduction.^14,15^ Indeed, In_2_O_3_ alloying has been utilized to increase mobility in heterostructure transistors and to effectively reduce the band gap to cover the entire solar-blind spectrum.^16,17^ In practice, the properties and stability of the  alloys may strongly depend on the preparation method, which include sol–gel,^18^ sputtering,^19^ and pulsed laser deposition.^20,21^ An in-depth exploration of the fundamental characteristics of these alloys is critical for understanding material growth, defect optimization, and overall device development to achieve the expected high performance of these emerging materials.

The majority of the experimental and theoretical studies of alloys between Ga_2_O_3_ and group-III element oxides focuses on , with several works focusing on the engineering of their band gap and growth mechanisms.^22−27^ Less attention has been paid to  alloys.^14,15,17,28−32^ Although indium, gallium, and aluminum are isovalent, their ambient stable oxides are not isomorphous due to different cation sizes. For instance, there are significant differences between the monoclinic β-Ga_2_O_3_ and body-centered cubic (BCC) In_2_O_3_ (bixbyite) structures, including lattice parameters and cation coordination. These differences yield a large miscibility gap for the  alloys.^33,34^

In this work, we comprehensively assess alloy structures of  with indium fraction *x* ∈ [0, 0.31] from first-principles to accurately predict the band gaps, effective masses, and their dependence on indium concentration and disorder.

The electronic structure of  alloys has been already studied from first-principles by Liu and Tan.^14^ In that study, semilocal functionals were employed, which are known to underestimate the band gap, and a supercell of 80 atoms was used, which is rather too small for modeling accurately the disordered structures.^27^ At variance with Liu and Tan,^14^ we use the Heyd, Scuseria, and Ernzerhof (HSE06) hybrid functional,^35,36^ which returns accurate band gaps without the need for empirical adjustments (e.g., by means of “scissor operators”). Also, we employ a larger 160-atom supercell with a special quasirandom structure to model accurately the alloys’ substitutional disorder.

At the variance of previous studies, we also report the effective band structure (EBS) of  alloys, which shows that the substitutional disorder has a minimal impact on the lower conduction band, with no defects introduced into the band gap. The band gap, *E*_g_, decreases linearly with the indium fraction, *x*, and is consistent with the **k**·**p** theory,^37^ as we observe a clear linear dependence of the electron inverse effective mass with 1/*E*_g_.

Finally, we investigate strain induced by the lattice mismatch, and we determine the critical epitaxial growth thickness, which is relevant to the growth of high-quality  films and for which an estimate was missing.

## Computational Details

First-principles simulations were carried out using the pseudopotential plane-wave code Vienna Ab initio Simulation Package (VASP).^38−40^ The generalized gradient approximation Perdew–Burke–Ernzerhof (GGA PBE),^41^ as well as the strongly constrained and appropriately normed (SCAN)^42^ exchange–correlation functionals were used. The band gap—which is underestimated by both PBE and SCAN functionals—was obtained using the HSE06 hybrid functional^35,36^ with a mixing parameter of 0.32, which was previously applied to both parent compounds and gives excellent agreement between the calculated and experimental band gap and lattice parameter.^24,26,34,43^ A Monkhorst–Pack sampling scheme of the supercells’ Brillouin zone was used, with a 0.03 1/Å separation. Electron-ion interactions were described using the projected augmented wave pseudopotential^44^ with a plane-wave basis set cutoff energy of 600 eV and valence configuration d^10^ s^2^ p^1^ for indium, d^10^ s^2^ p^1^ for gallium, and s^2^ p^4^ for oxygen. Including the d-states is crucial for obtaining accurate results.^24^ The structures were relaxed until the maximum force component was smaller than 0.02 eV/Å and the energy tolerance was set to 10^–6^ eV.

The EBS is computed using the standard band unfolding scheme,^45−47^ to facilitate a direct comparison between the supercell (SC) and primitive cell (PC) band structures. As the PC and SC are commensurate, we have , with *i* = 1, ..., *N*, where **K⃗** is a wave vector of the SC,  is a wave vector of the PC, and  is a reciprocal lattice vector of the SC. Here, *N* is the ratio of the volumes of the PC and SC Brillouin zones. Band unfolding is based on the expression of SC eigenvectors as a linear combination of PC eigenvectors. The spectral weight, defined as
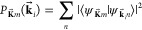
1determines the probability of an SC eigenstate to have the same Bloch character as a PC eigenstate.^27^ From the knowledge of the spectral weight, the spectral function
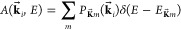
2is obtained, where  is the energy of the SC eigenvector . The EBS is given by the spectral function and shown in Figure 3.

## Results and Discussion

### Disorder and the Preferential Site

First, we optimize the conventional cell of β-Ga_2_O_3_ and use supercell software^48^ to create the alloy structures. A complete set of alloy SCs can be generated by considering all of the permutations of indium and gallium atoms. This set contains numerous structures, and their number can be reduced by retaining only the symmetry-inequivalent ones. We assume the monoclinic phase for all considered In concentrations, which is expected for Ga-rich alloys.^34^ The SC approach was selected instead of the less computationally demanding virtual crystal approximation since in our tests, the latter failed to reproduce the trends experimentally observed for either  or  alloys.

For each indium fraction considered, we initially screened the SC total energies using 40-atom SCs without constraining the site occupations, i.e., by exhausting all of the random indium substitutions. Our simulations predict that in the  alloys, the indium atoms preferably occupy the octahedral sites, analogously to what we observed for  alloys (not shown) and in agreement with previous studies of both aluminum or indium alloys^25−27^ (though as an exception, ref (23) reports preferential occupation of the tetrahedral sites).

**Figure 1:**
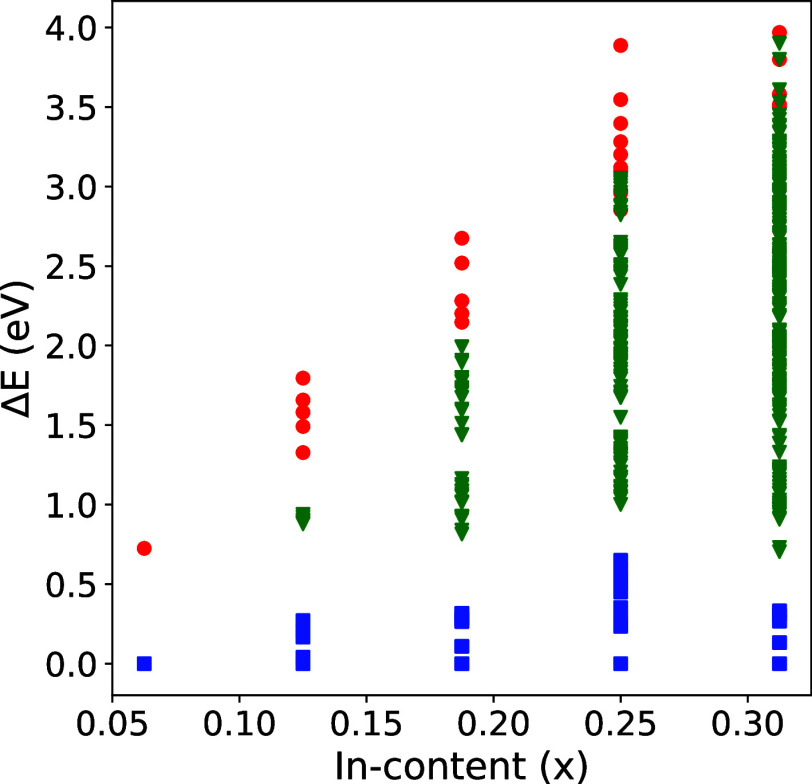
Total energy differences for β- alloy structures as a function of the In fraction, *x*. The lowest energy for each indium fraction is set as the reference (i.e., to 0 eV). Blue squares, red circles, and green triangles represent octahedral, tetrahedral, and mixed occupational configurations, respectively. A few low-lying configurations in all cases are separated by approximately 0.1 eV.

Figure 1 shows the dispersion of the total energy of the 40-atom SCs with respect to the fraction of indium atoms in the alloy. The total energy of the most energetically favorable configuration is set as the reference. Clustering of the SCs according to the indium site occupation along with a large difference between the octahedral and tetrahedral site occupation is evident from the figure.

At low indium fraction, the monoclinic structure is preferable, and the substituted indium atoms avoid configurations in which they sit in the nearest neighbor octahedral sites. As the indium concentration increases, all second-nearest-neighbor sites get occupied, forcing the additional indium atoms to occupy shared-edge octahedra. In general, compared with the energy difference between occupation of tetrahedral and octahedral sites, the energy difference between the occupied octahedral sites is relatively smaller. At higher concentrations, the octahedral sites become fully occupied, and additional indium atoms eventually occupy the tetrahedral sites, too. It should be noted that at higher indium fraction, the monoclinic structure is no longer favorable, and it does not make more sense to distinguish between octahedral and tetrahedral sites. However, in the current work, we only focus on gallium-rich conditions where the monoclinic structure is still preferable.

Given the preferential occupation of the octahedral sites, we increased the SC size to 1 × 2 × 2 and constrained the indium occupation by avoiding tetrahedral site occupation. All possible indium substitutions within octahedral sites were considered, and the most energetically favorable configuration for each indium fraction, *x*, was selected.

**Figure 2:**
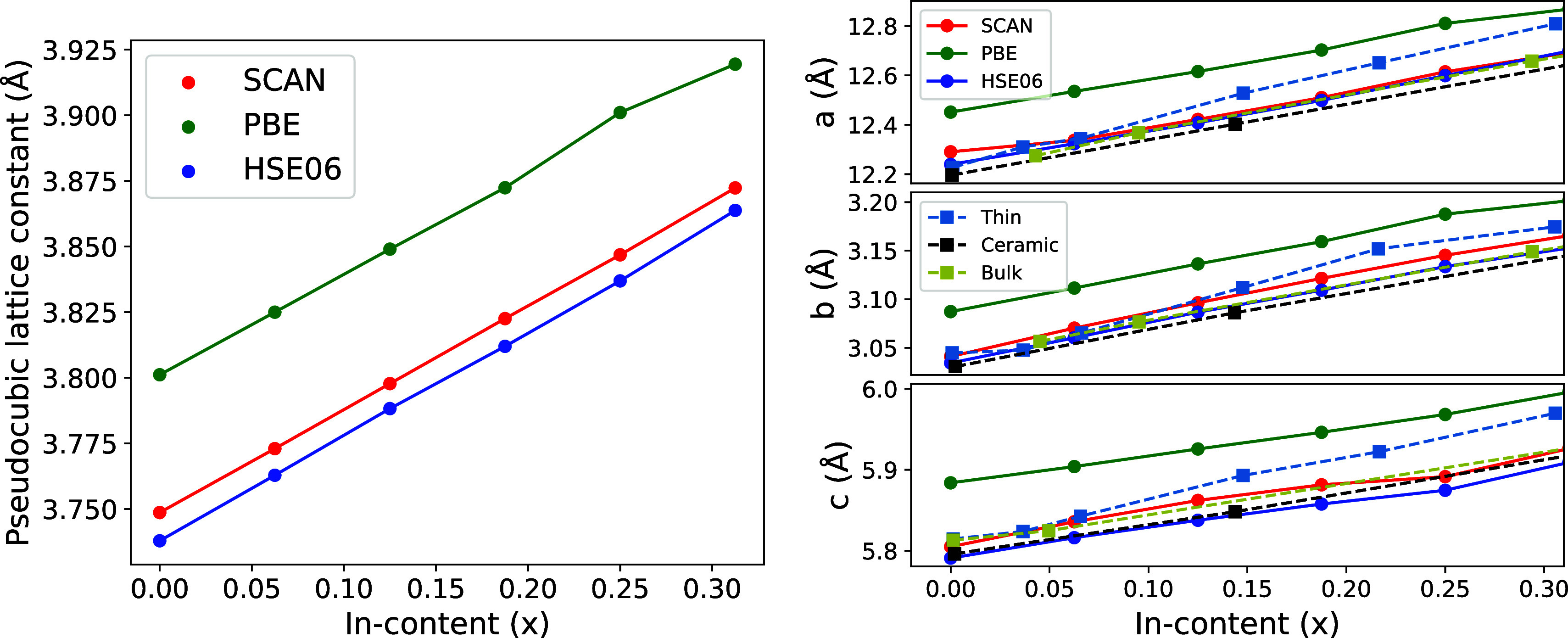
Pseudocubic and crystal lattice constants of β- alloys as a function of fraction of indium atoms, *x*. Reference experimental data are obtained from the literature.^18,32,33^ Squares represent bulk powder, thin film, and ceramic samples. Results using either SCAN or HSE06 functionals are quite close, while PBE slightly overestimates the lattice constants. In general, the trend is linear with a very similar slope in all cases.

Figure 2 displays the lattice parameters, as well as the pseudocubic lattice constant, defined as , where *V* is the optimized volume of the unit cell. As reported in previous studies,^14,32^ a clear linear trend (Vegard’s law) with the indium fraction, *x*, is found. The slope is almost independent of the functional used. Both SCAN and HSE06 functionals provide very similar lattice parameters, and agree well with previously reported experimental results for ceramic (polycrystalline)^32^ and bulk.^33^ Systematic deviation is observed for thin films.^18^ This behavior is likely due to the larger ionic radius of indium atoms compared to gallium atoms and the strain possibly present in thin films.

The special quasirandom structure (SQS) approach has been used to provide a more efficient approach for larger 2 × 2 × 2 SCs containing 160 atoms. In these SCs, clusters composed of two or three atoms are used to compare the site occupancy correlations of SQSs against truly random alloy structures. Monte Carlo sampling, as implemented in the mcsqs code distributed with the Alloy Theoretic Automated Toolkit (ATAT),^49^ is used to minimize the correlation mismatch for all indium fractions considered.

### Effective Band Structure

Band unfolding is used to obtain the EBS of the  alloys. As previously reported,^27^ the use of small SCs can create artificially induced states in the effective conduction bands. We found that an SC with >100 atoms is necessary to avoid such spurious states. In this study, we report results for large 2 × 2 × 2 SCs (160 atoms) generated using the SQS approach.

Because of the system size, the EBS was obtained at the PBE level. An accurate band gap was then calculated at the HSE level from a nonself-consistent calculation on a few **k**-points around the Γ point. As previously discussed for similar materials,^27^ the PBE band structure is expected to be reasonably accurate, apart from the band gap underestimation. For instance, the band curvature given by PBE is expected to closely agree with that of HSE06.^27^ The spectral function of the EBS, which provides a qualitative measure of Bloch’s character conserved after the band unfolding, is computed using eq 1.

**Figure 3:**
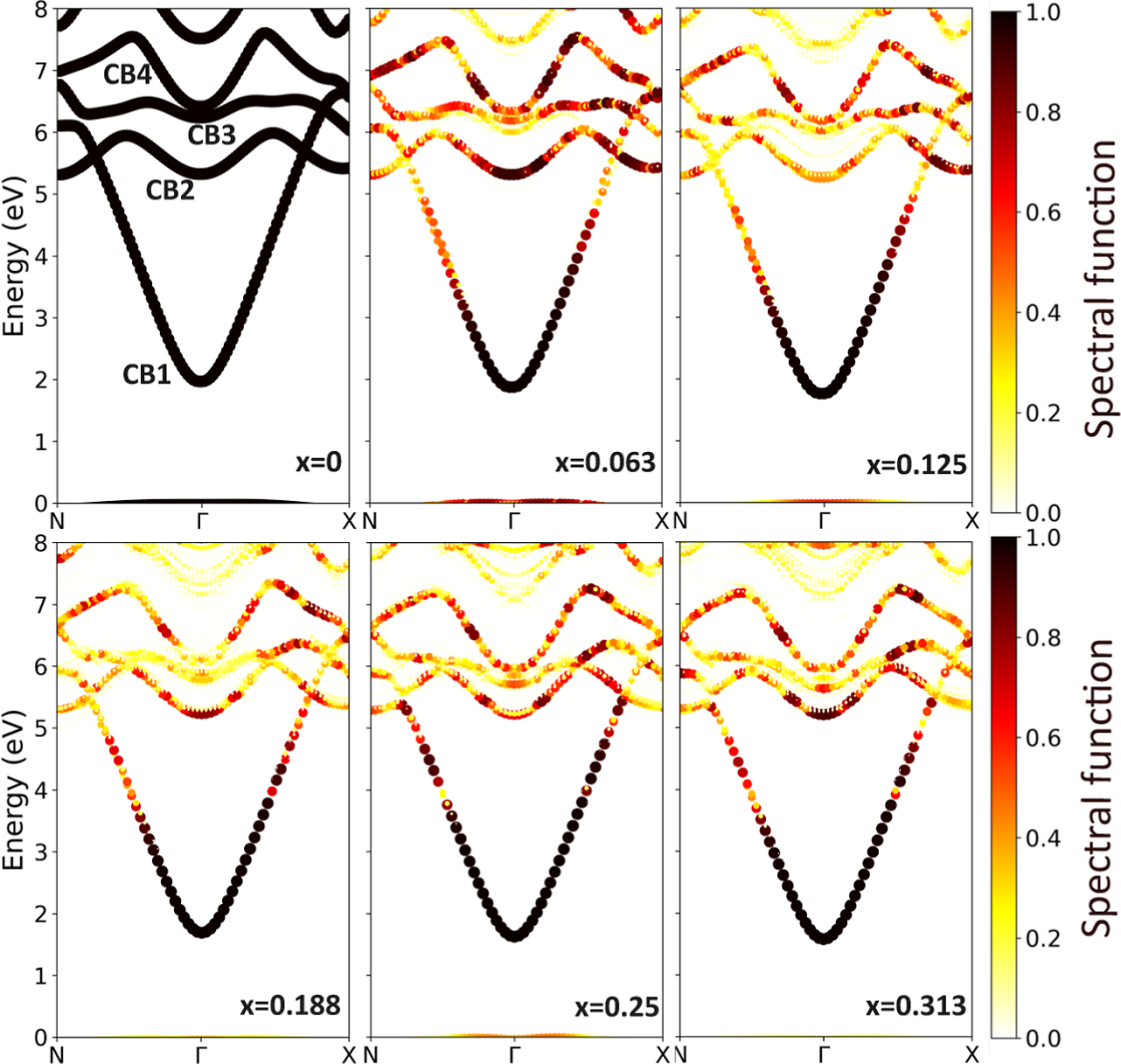
EBSs at the PBE level for β-(In_*x*_Ga_1–*x*_)_2_O_3_ alloys with *x* = 0.0625, 0.125, 0.1875, 0.25, and 0.3125 using a 160-atom SC. The valence band maximum (VBM) is set to zero. The color bar shows the calculated spectral function, which quantifies the Bloch character conserved in the SC.

In Figure 3, the EBS of  is reported. The lower conduction band, especially in the regions surrounding the conduction band minimum (CBM), is mostly unaffected by disorder for all of the indium fractions. No impurity states were observed in the band gap of these alloys, in contrast with GaN_1–x_P_*x*_. In that case, even a minor phosphorus substitution yields a strongly localized (t_2_-like) level that evolves into an impurity band as the phosphorus fraction is increased.^45,46^

Higher energy conduction bands of  are more affected by indium substitution, as evidenced by the larger variation of their spectral functions, rendered in Figure 3 as a broadening of the band structure. In particular, the conduction band named “CB3” almost disappears as the indium fraction, *x*, is increased. This is due to the broken translational symmetry in the alloys. The broadening can be also used to characterize the alloy scattering rate and electron mobility, as previously reported for Al_*x*_Ga_1–*x*_N alloys.^50^ All bands exhibit high isotropy around the Brillouin zone center, a characteristic inherited from the parent compound, β-Ga_2_O_3_.

**Figure 4:**
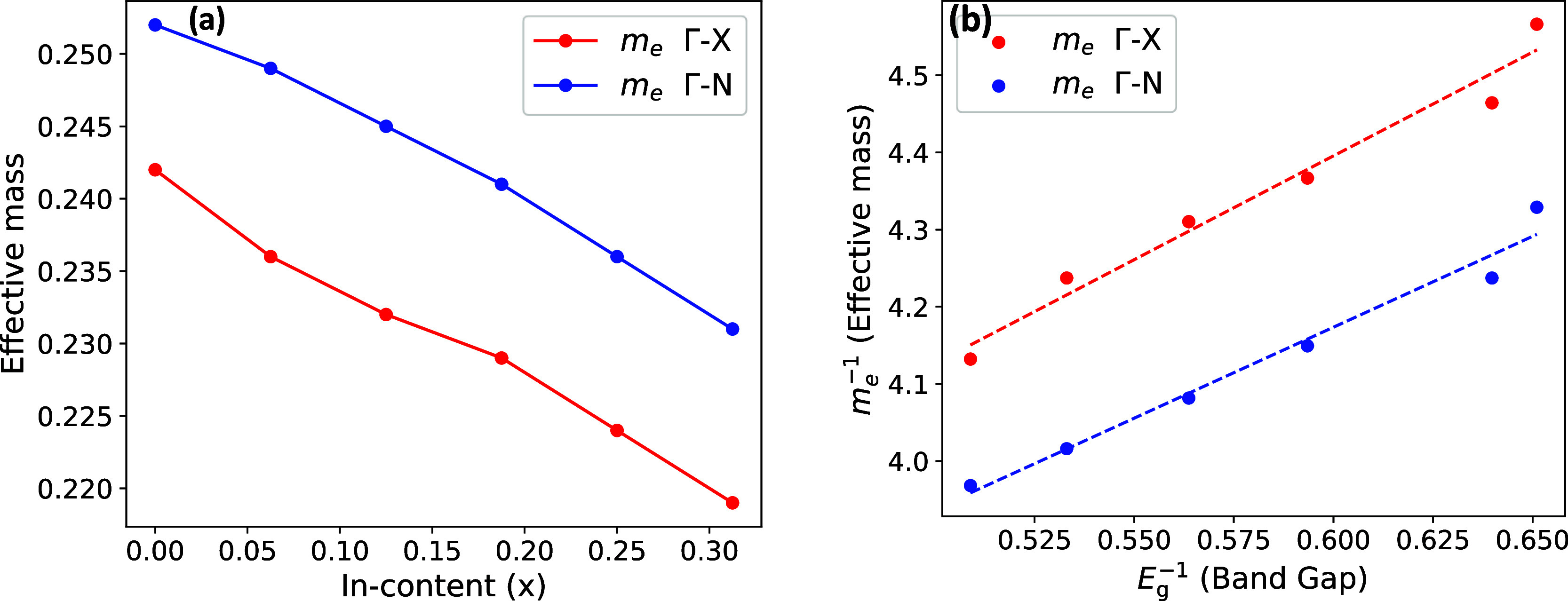
(a) Calculated effective mass for  alloys as a function of In content (*x*). The effective mass was determined using the PBE functional. (b) Inverse electron effective mass, *m*_e_^–1^, as a function of *E*_g_^–1^, with dashed lines indicating the best linear fit.

Using the parabolic approximation at the band edge, we computed the electron effective mass along the Γ–*X* and Γ–*N* directions. In Figure 4a, the electron effective masses are reported as a function of the indium fraction, *x*. For the pristine β-Ga_2_O_3_, our calculated effective masses are in good agreement with those experimentally reported (*m*_e_ ≈ 0.28).^51^ Furthermore, in Figure 4b, a linear relationship is observed between the inverse electron effective mass, *m*_e_^–1^, and *E*_g_^–1^, as expected from the **k**·**p** perturbation theory.^52^ At variance with previous studies,^14^ which show a rather constant electron effective mass, we found that the conduction electron effective masses follow a linear trend very similar to that shown by the lattice parameters and the band gaps, as seen in Figure 4. This is likely due to the S size (80 atoms) used in previous studies, which is not sufficient to accurately model the substitutional disorder. As shown by Sharma et al.^27^ for  alloys, when a larger SC is used, a clear linear trend of the electron effective mass is observed. The valence band maximum is almost flat (Figure S4), suggesting a large effective mass for the holes. Far from the valence band maximum, a small hole effective mass of 0.32*m*_e_ is determined along the Γ–*A* direction. This observation aligns with the previous results.^10^ A large effective hole mass can yield polaronic-like transport if the holes become self-trapped by lattice distortions. This is indeed a common feature of semiconductor oxides.^53^

**Figure 5:**
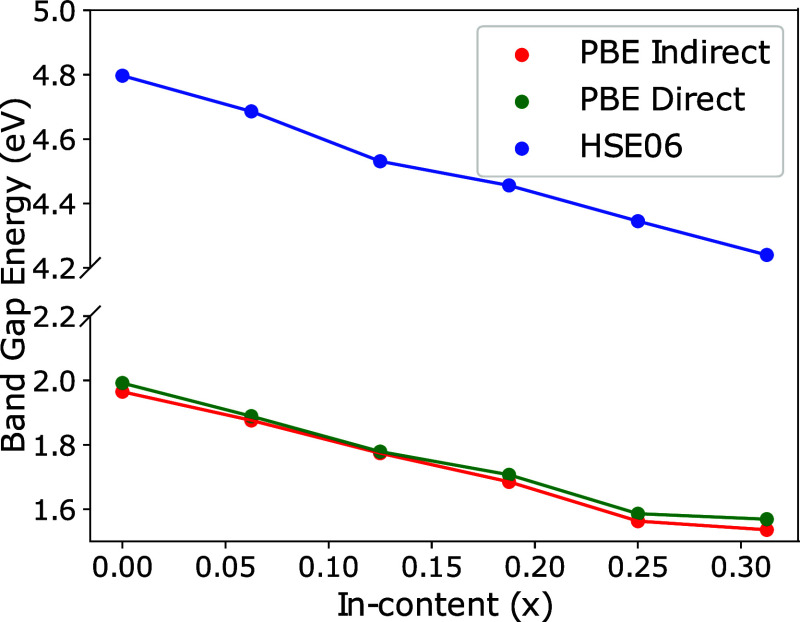
Band gap of  alloys as functions of the indium fraction, *x*. Indirect band gaps calculated using the HSE06 functional are in good agreement with the experimental values (see text). Direct and indirect band gaps calculated using the PBE functional are reported for comparison.

Figure 5 shows the band gap dependence on the indium fraction, *x*. For all values of *x* considered in this study, the CBM is located at the Γ point. Because of the small dispersion of the highest valence band, determining accurately the VBM is nontrivial. Indeed, the difference between the minimum direct and indirect band gaps is smaller than 0.04 eV. All HSE06 band gaps presented in Figure 4 are indirect. Our findings agree with the previous reports,^14,27^ suggesting that the minimum band gap is indirect for both aluminum and indium alloys.

### Surface Energy and Critical Thickness

Five slabs of both β-Ga_2_O_3_ and monoclinic In_2_O_3_ have been generated to expose the (100)A, (100)B, (010), (001)A, and (001)B surfaces, respectively (see Figure S2). The corresponding surface energies, γ_100A_, γ_100B_, γ_010_, γ_001A_, and γ_001B_, are reported in Table 1. Convergence of the surface energy has been ensured by comparing slabs of increasing thickness. As the slab’s total energy depends linearly on the thickness, the surface energy was computed using linear interpolation (computational details are presented in the Supporting Information). In general, surface energies are lower than group-III nitride materials,^56^ which suggests enhanced stability of these surfaces. The same trend has been observed for both β-Ga_2_O_3_ and monoclinic In_2_O_3_ surface energies, namely, γ_100B_ < γ_100A_ < γ_001B_ < γ_010_ < γ_001A_. Our results determined using the SCAN functional show good agreement with previous HSE06 calculations,^26^ while being less computationally demanding than a hybrid functional. In line with previous studies,^26^ the surface energies of  are obtained using linear interpolation between the pure phases In_2_O_3_ and β-Ga_2_O_3_.

**Table 1:** Calculated Unrelaxed Surface Energies (in J/m^2^) of β-Ga_2_O_3_ and Monoclinic In_2_O_3_ Surfaces for [100], [010], and [001] Orientations (Also See Figure S2)^a^

surfaces	β-Ga_2_O_3_				monoclinic-In_2_O_3_
	this work (SCAN)	HSE06^26^	DFT^54^	DFT^55^	this work (SCAN)
100A	1.66	1.39	1.68	1.28	1.35
100B	0.91	0.60	0.96	0.61	0.79
010	2.46	2.52	2.78	2.23	1.95
001B	2.35	2.37	2.65		1.92
001A	2.82	2.98	3.35		2.07

a Only symmetric and nonpolar terminations are considered. Previously published results are listed for comparison.

In pseudomorphic growth, both substrate and film have the same crystallographic structure. In this study, we assume that the β- alloys are epitaxially grown on a β-Ga_2_O_3_ surface with one of the orientations listed above. The structural misfit can be controlled by alloying, as increasing the indium fraction, *x*, stretches the lattice parameters of the epilayer. Such stretching strains the epilayer, accumulating elastic energy until a critical value is reached and the formation of lattice defects becomes energetically favorable. We first compute the elastic constants, *C*_*ij*_, of both β-Ga_2_O_3_ and the  alloys. The obtained values of *C*_*ij*_ for β-Ga_2_O_3_ show good agreement with previously calculated and experimental values (see Supporting Information Table S1).^26,57^ Next, for each surface orientation, we compute the strain, ϵ_*i*_, due to the lattice mismatch (Table S2 in the Supporting Information). The in-plane components of the strain tensor are computed directly using the ratio between the film and substrate lattice constants. The other components are computed by minimizing the elastic energy, .

Given the surface energy, strains, and elastic constants, the critical thickness, *h*_c_, can be determined from Griffith’s theory^58,59^
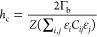
3where the sum is over strain, ε_*i*_, and elastic constants, *C*_*ij*_, components. Γ_b_ is the brittle fracture toughness and *Z* is a dimensionless factor that depends on the crack type (*Z* = 1.976 for the channelling type^59^).

Compared to , a larger strain is found for  due to the greater lattice mismatch between the parent oxides. As a consequence, large elastic energy is stored in the growing film, leading to a lower critical thickness. The plane with the lowest surface energy is assumed to be the crack plane. Here, we consider the surface energy of the unrelaxed crack plane since this corresponds to half of the energy needed to cleave the bulk crystal.^26,60^

According to our unrelaxed surface calculations, (100)B is identified as having a lower surface energy, making it the preferential crack plane for both [010] and [001] film growth orientations. For the [100] orientation, the corresponding crack plane is (001)B, given that its surface energy is lower than that of (010).

**Figure 6:**
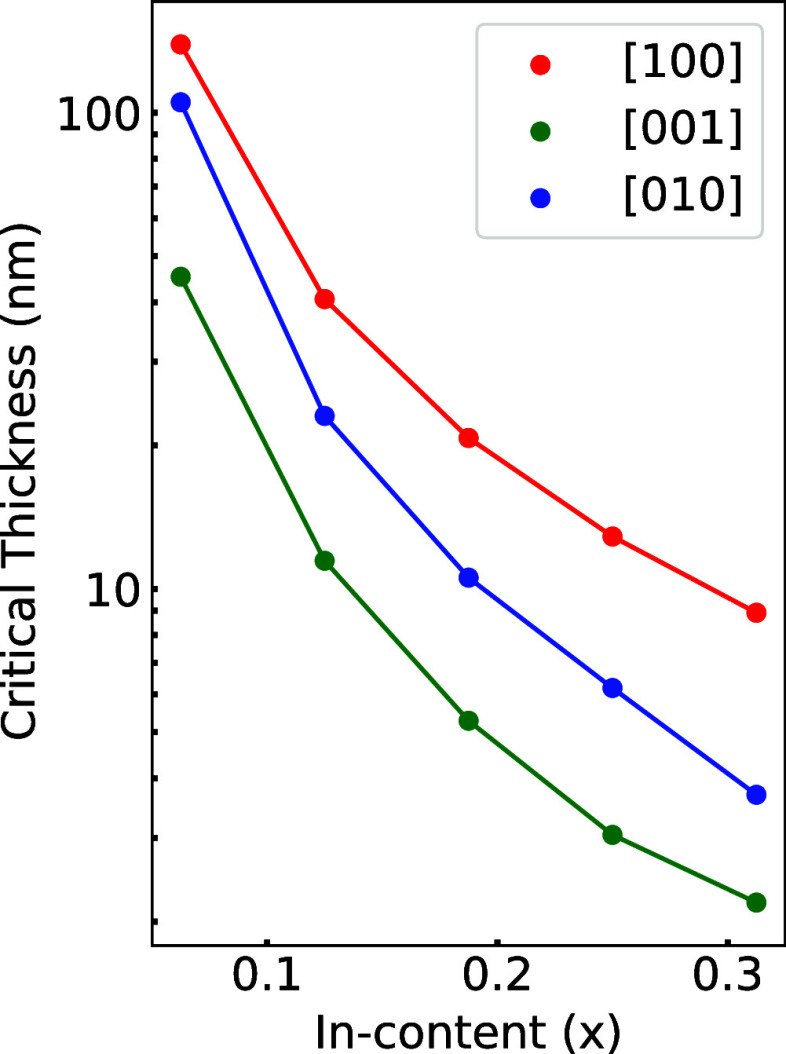
Critical thickness (*h*_c_, in nm) for  alloys epitaxially grown on β-Ga_2_O_3_ surfaces as a function of indium fraction, *x*. *h*_c_ is shown for (100) (red), (010) (blue), and (001) (green) plane growth.

Figure 6 presents the critical thickness, *h*_c_, as a function of the indium fraction, *x*. The [100]-grown films are less likely to crack, as they display the largest value of *h*_c_. This is mainly due to the higher surface energy associated with the crack plane (2.35 J/m^2^). Conversely, since the (100)B surface has a lower surface energy (0.91 J/m^2^), during the growth of [010] and [001] films, the (100)B surface is expected to crack, showing a lower critical thickness. In general, the values of *h*_c_ found for  are smaller than those of nitrides (GaN, AlN) and  alloys^56^ due to the larger lattice mismatch as well as lower surface energies. In practice, the critical thicknesses calculated in this study should be understood as lower bounds, since we assumed brittle fracture instead of considering fracture toughness where plastic deformation occurs.^56^

## Conclusions

In conclusion, we conducted accurate first-principles investigation of monoclinic  alloys for *x* ∈ [0, 0.31]. Indium atoms predominantly occupy octahedral sites, which are more energetically favorable than tetrahedral sites. The lattice constants increase linearly with the indium fraction, *x*, in agreement with the experimental observations. The band gap, determined at the HSE06 level, is indirect for all indium fractions considered. A linear decrease in the band gap, from 4.8 to 4.24 eV, is observed. We have reported the EBS of  alloys and observed that the lower conduction band remains parabolic and qualitatively unaffected by the substitutional disorder. The electron effective mass is well defined and also decreases linearly with the indium fraction. The hole effective mass cannot be determined confidently due to the flatness of the valence bands. No defect states were observed in the gap. Finally, we reported the surface energies and strains for pseudomorphic epitaxial growth of  alloys on β-Ga_2_O_3_ surfaces along the [100], [010], and [001] orientations. By employing Griffith’s theory, we provide estimates of the critical thickness, *h*_c_, of  alloys epitaxially grown on β-Ga_2_O_3_. Our estimates of *h*_c_ are generally smaller than the critical thickness for nitrides (GaN, AlN) and  alloys.

## Data Availability

The data underlying this study are available in the published article and its Supporting Information.

## Supporting Information Available

Detailed information on modeling, including surface energy and *k*-point convergence; structures employed for modeling in both disordered and surface systems; and EBS theoretical details, computed elastic constants, stability, and strain components for growth scenarios along [100], [010], and [001] (PDF)

## References

[ref1] Higashiwaki M.; Jessen G. H. Guest Editorial: The dawn of gallium oxide microelectronics. Appl. Phys. Lett. 2018, 112, 060401 10.1063/1.5017845.

[ref2] Kong J.; Nordlund D.; Jin J. S.; Kim S. Y.; Jin S. M.; Huang D.; Zheng Y.; Karpovich C.; Sertic G.; Wang H.; et al. Underwater Organic Solar Cells via Selective Removal of Electron Acceptors near the Top Electrode. ACS Energy Lett. 2019, 4, 1034–1041. 10.1021/acsenergylett.9b00274.

[ref3] Higashiwaki M.; Sasaki K.; Kuramata A.; Masui T.; Yamakoshi S. Gallium oxide (Ga2O3) metal-semiconductor field-effect transistors on single-crystal β-Ga2O3 (010) substrates. Appl. Phys. Lett. 2012, 100, 013504 10.1063/1.3674287.

[ref4] Hwang W. S.; Verma A.; Peelaers H.; Protasenko V.; Rouvimov S.; Grace Xing H.; Seabaugh A.; Haensch W.; de Walle C. V.; Galazka Z.; Albrecht M.; Fornari R.; Jena D. High-voltage field effect transistors with wide-bandgap β-Ga2O3 nanomembranes. Appl. Phys. Lett. 2014, 104, 203111 10.1063/1.4879800.

[ref5] Roy R.; Hill V. G.; Osborn E. F. Polymorphism of Ga2O3 and the System Ga2O3—H2O. J. Am. Chem. Soc. 1952, 74, 719–722. 10.1021/ja01123a039.

[ref6] Zinkevich M.; Aldinger F. Thermodynamic Assessment of the Gallium-Oxygen System. J. Am. Ceram. Soc. 2004, 87, 683–691. 10.1111/j.1551-2916.2004.00683.x.

[ref7] Víllora E. G.; Shimamura K.; Yoshikawa Y.; Aoki K.; Ichinose N. Large-size β-Ga2O3 single crystals and wafers. J. Cryst. Growth 2004, 270, 420–426. 10.1016/j.jcrysgro.2004.06.027.

[ref8] Aida H.; Nishiguchi K.; Takeda H.; Aota N.; Sunakawa K.; Yaguchi Y. Growth of β-Ga_2_O_3_ Single Crystals by the Edge-Defined, Film Fed Growth Method. Jpn. J. Appl. Phys. 2008, 47, 8506 10.1143/JJAP.47.8506.

[ref9] Åhman J.; Svensson G.; Albertsson J. A Reinvestigation of β-Gallium Oxide. Acta Crystallogr., Sect. C: Cryst. Struct. Commun. 1996, 52, 1336–1338. 10.1107/S0108270195016404.

[ref10] Varley J. B.; Weber J. R.; Janotti A.; Van De Walle C. G. Oxygen vacancies and donor impurities in β-Ga2O3. Appl. Phys. Lett. 2010, 97, 142106 10.1063/1.3499306.

[ref11] Varley J. B.; Peelaers H.; Janotti A.; Van de Walle C. G. Hydrogenated cation vacancies in semiconducting oxides. J. Phys.: Condens. Matter 2011, 23, 334212 10.1088/0953-8984/23/33/334212. 21813965

[ref12] Lyons J. L. A survey of acceptor dopants forβ-Ga_2_O_3_. Semicond. Sci. Technol. 2018, 33, 05LT02 10.1088/1361-6641/aaba98.

[ref13] Kyrtsos A.; Matsubara M.; Bellotti E. On the feasibility of p-type Ga2O3. Appl. Phys. Lett. 2018, 112, 032108 10.1063/1.5009423.

[ref14] Liu X.; Tan C.-K. Electronic properties of monoclinic (InxGa1-x)2O3 alloys by first-principle. AIP Adv. 2019, 9, 035318 10.1063/1.5093195.

[ref15] Lee H. U.; Kim H. W.; Fatti G.; Ko H.; Cho S. B. Dominant Effects of Epitaxial Strain on the Phase Control of Heterostructural (In _x_ Ga _1–x_) _2_ O _3_ Alloys. ACS Appl. Electron. Mater. 2022, 4, 2711–2717. 10.1021/acsaelm.2c00240.

[ref16] Zhang Z.; von Wenckstern H.; Lenzner J.; Lorenz M.; Grundmann M. Visible-blind and solar-blind ultraviolet photodiodes based on (InxGa1-x)2O3. Appl. Phys. Lett. 2016, 108, 123503 10.1063/1.4944860.

[ref17] Fares C.; Xian M.; Smith D. J.; McCartney M. R.; Kneiß M.; Von Wenckstern H.; Grundmann M.; Tadjer M.; Ren F.; Pearton S. J. Changes in band alignment during annealing at 600 °C of ALD Al2O3 on (InxGa1 - x)2O3 for x = 0.25–0.74. J. Appl. Phys. 2020, 127, 105701 10.1063/5.0002875.

[ref18] Kokubun Y.; Abe T.; Nakagomi S. Sol–gel prepared (Ga_1–x_In_x_)_2_O_3_ thin films for solar-blind ultraviolet photodetectors. Phys. Status Solidi A 2010, 207, 1741–1745. 10.1002/pssa.200983712.

[ref19] Lu H.; Dong H.; Jiao S.; Nie Y.; Wang X.; Wang D.; Gao S.; Wang J.; Su S. The Influence of Annealing Temperature on Structure, Morphology and Optical Properties of (InxGa1-x)2O3 Films. ECS J. Solid State Sci. Technol. 2019, 8, Q3171–Q3175. 10.1149/2.0311907jss.

[ref20] Wenckstern H. v.; Splith D.; Purfürst M.; Zhang Z.; Kranert C.; Müller S.; Lorenz M.; Grundmann M. Structural and optical properties of (In, Ga) 2O3 thin films and characteristics of Schottky contacts thereon. Semicond. Sci. Technol. 2015, 30, 024005 10.1088/0268-1242/30/2/024005.

[ref21] Fares C.; Kneiß M.; von Wenckstern H.; Grundmann M.; Tadjer M.; Ren F.; Lambers E.; Pearton S. Valence band offsets for ALD SiO2 and Al2O3 on (InxGa1- x) 2O3 for x= 0.25–0.74. APL Mater. 2019, 7, 071115 10.1063/1.5110498.

[ref22] Krueger B. W.; Dandeneau C. S.; Nelson E. M.; Dunham S. T.; Ohuchi F. S.; Olmstead M. A. Variation of Band Gap and Lattice Parameters of β-(Al _x_ Ga _1-x_) _2_ O _3_ Powder Produced by Solution Combustion Synthesis. J. Am. Ceram. Soc. 2016, 99, 2467–2473. 10.1111/jace.14222.

[ref23] Li J.; Chen X.; Ma T.; Cui X.; Ren F.-F.; Gu S.; Zhang R.; Zheng Y.; Ringer S. P.; Fu L.; Tan H. H.; Jagadish C.; Ye J. Identification and modulation of electronic band structures of single-phase β-(AlxGa1–x)2O3 alloys grown by laser molecular beam epitaxy. Appl. Phys. Lett. 2018, 113, 041901 10.1063/1.5027763.

[ref24] Peelaers H.; Varley J. B.; Speck J. S.; Van De Walle C. G. Structural and electronic properties of Ga2O3-Al2O3 alloys. Appl. Phys. Lett. 2018, 112, 242101 10.1063/1.5036991.

[ref25] Ratnaparkhe A.; Lambrecht W. R. L. Quasiparticle Self-Consistent *GW* Study of (Ga_1–x_Al_x_)_2_O_3_ Alloys in Monoclinic and Corundum Structures. Phys. Status Solidi B 2020, 257, 1900317 10.1002/pssb.201900317.

[ref26] Mu S.; Wang M.; Peelaers H.; Van De Walle C. G. First-principles surface energies for monoclinic Ga2O3 and Al2O3 and consequences for cracking of (Al *x* Ga1- *x*)2O3. APL Mater. 2020, 8, 091105 10.1063/5.0019915.

[ref27] Sharma A.; Singisetti U. Effective electronic band structure of monoclinic β-(AlxGa1-x)2O3 alloy semiconductor. AIP Adv. 2023, 13, 015101 10.1063/5.0134155.

[ref28] Papadogianni A.; Wouters C.; Schewski R.; Feldl J.; Lähnemann J.; Nagata T.; Kluth E.; Feneberg M.; Goldhahn R.; Ramsteiner M.; Albrecht M.; Bierwagen O. Molecular beam epitaxy of single-crystalline bixbyite (In 1 - x Ga x) 2 O 3 films (x *a* ≤ *b* 0.18): Structural properties and consequences of compositional inhomogeneity. Phys. Rev. Mater. 2022, 6, 033604 10.1103/PhysRevMaterials.6.033604.

[ref29] Maccioni M. B.; Fiorentini V. Phase diagram and polarization of stable phases of (Ga _1–x_ In _x_) _2_ O _3_. Appl. Phys. Express 2016, 9, 041102 10.7567/APEX.9.041102.

[ref30] Nishinaka H.; Miyauchi N.; Tahara D.; Morimoto S.; Yoshimoto M. Incorporation of indium into ε -gallium oxide epitaxial thin films grown *via* mist chemical vapour deposition for bandgap engineering. CrystEngComm 2018, 20, 1882–1888. 10.1039/C7CE02103H.

[ref31] Peelaers H.; Steiauf D.; Varley J. B.; Janotti A.; Van De Walle C. G. (In x Ga 1 - x) 2 O 3 alloys for transparent electronics. Phys. Rev. B 2015, 92, 085206 10.1103/PhysRevB.92.085206.

[ref32] Kranert C.; Lenzner J.; Jenderka M.; Lorenz M.; Von Wenckstern H.; Schmidt-Grund R.; Grundmann M. Lattice parameters and Raman-active phonon modes of (In *x* Ga1–x)2O3 for x 0.4. J. Appl. Phys. 2014, 116, 013505 10.1063/1.4886895.

[ref33] Edwards D. D.; Folkins P. E.; Mason T. O. Phase Equilibria in the Ga2O3In2O3 System. J. Am. Ceram. Soc. 1997, 80, 253–257. 10.1111/j.1151-2916.1997.tb02820.x.

[ref34] Swallow J. E. N.; Palgrave R. G.; Murgatroyd P. A. E.; Regoutz A.; Lorenz M.; Hassa A.; Grundmann M.; Von Wenckstern H.; Varley J. B.; Veal T. D. Indium Gallium Oxide Alloys: Electronic Structure, Optical Gap, Surface Space Charge, and Chemical Trends within Common-Cation Semiconductors. ACS Appl. Mater. Interfaces 2021, 13, 2807–2819. 10.1021/acsami.0c16021. 33426870

[ref35] Heyd J.; Scuseria G. E.; Ernzerhof M. Hybrid functionals based on a screened Coulomb potential. J. Chem. Phys. 2003, 118, 8207–8215. 10.1063/1.1564060.

[ref36] Krukau A. V.; Vydrov O. A.; Izmaylov A. F.; Scuseria G. E. Influence of the exchange screening parameter on the performance of screened hybrid functionals. J. Chem. Phys. 2006, 125, 224106 10.1063/1.2404663. 17176133

[ref37] Jaros M. Electronic properties of semiconductor alloy systems. Rep. Prog. Phys. 1985, 48, 1091–1154. 10.1088/0034-4885/48/8/001.

[ref38] Kresse G.; Furthmüller J. Efficient iterative schemes for ab initio total-energy calculations using a plane-wave basis set. Phys. Rev. B 1996, 54, 11169–11186. 10.1103/PhysRevB.54.11169. 9984901

[ref39] Kresse G.; Furthmüller J. Efficiency of ab-initio total energy calculations for metals and semiconductors using a plane-wave basis set. Comput. Mater. Sci. 1996, 6, 15–50. 10.1016/0927-0256(96)00008-0. 9984901

[ref40] Kresse G.; Hafner J. Ab initio molecular dynamics for liquid metals. Phys. Rev. B 1993, 47, 558–561. 10.1103/PhysRevB.47.558. 10004490

[ref41] Perdew J. P.; Burke K.; Ernzerhof M. Generalized Gradient Approximation Made Simple. Phys. Rev. Lett. 1996, 77, 3865–3868. 10.1103/PhysRevLett.77.3865. 10062328

[ref42] Sun J.; Ruzsinszky A.; Perdew J. Strongly Constrained and Appropriately Normed Semilocal Density Functional. Phys. Rev. Lett. 2015, 115, 036402 10.1103/PhysRevLett.115.036402. 26230809

[ref43] Seacat S.; Lyons J. L.; Peelaers H. Properties of orthorhombic Ga2O3 alloyed with In2O3 and Al2O3. Appl. Phys. Lett. 2021, 119, 042104 10.1063/5.0060801.

[ref44] Blöchl P. E. Projector augmented-wave method. Phys. Rev. B 1994, 50, 17953–17979. 10.1103/PhysRevB.50.17953. 9976227

[ref45] Popescu V.; Zunger A. Effective Band Structure of Random Alloys. Phys. Rev. Lett. 2010, 104, 236403 10.1103/PhysRevLett.104.236403. 20867256

[ref46] Popescu V.; Zunger A. Extracting $E$ versus $\stackrel{P\vec}{k}$ effective band structure from supercell calculations on alloys and impurities. Phys. Rev. B 2012, 85, 085201 10.1103/PhysRevB.85.085201.

[ref47] Boykin T. B.; Kharche N.; Klimeck G.; Korkusinski M. Approximate bandstructures of semiconductor alloys from tight-binding supercell calculations. J. Phys.: Condens. Matter 2007, 19, 036203 10.1088/0953-8984/19/3/036203.

[ref48] Okhotnikov K.; Charpentier T.; Cadars S. Supercell program: a combinatorial structure-generation approach for the local-level modeling of atomic substitutions and partial occupancies in crystals. J. Cheminf. 2016, 8, 17 10.1186/s13321-016-0129-3. PMC4818540 27042215

[ref49] van de Walle A.; Tiwary P.; de Jong M.; Olmsted D. L.; Asta M.; Dick A.; Shin D.; Wang Y.; Chen L. Q.; Liu Z. K. Efficient stochastic generation of special quasirandom structures. Calphad 2013, 42, 13–18. 10.1016/j.calphad.2013.06.006.

[ref50] Pant N.; Deng Z.; Kioupakis E. High electron mobility of AlxGa1- xN evaluated by unfolding the DFT band structure. Appl. Phys. Lett. 2020, 117, 117 10.1063/5.0027802.

[ref51] Mohamed M.; Janowitz C.; Unger I.; Manzke R.; Galazka Z.; Uecker R.; Fornari R.; Weber J. R.; Varley J. B.; Van de Walle C. G. The electronic structure of β-Ga2O3. Appl. Phys. Lett. 2010, 97, 211903 10.1063/1.3521255.

[ref52] Jaros M. Electronic properties of semiconductor alloy systems. Rep. Prog. Phys. 1985, 48, 1091–1154. 10.1088/0034-4885/48/8/001.

[ref53] Speck J. S.; Farzana E. Ultrawide Bandgap β-Ga2O3 Semiconductor: Theory and Applications, 2023.

[ref54] Bermudez V. M. The structure of low-index surfaces of β-Ga2O3. Chem. Phys. 2006, 323, 193–203. 10.1016/j.chemphys.2005.08.051.

[ref55] Hinuma Y.; Gake T.; Oba F. Band alignment at surfaces and heterointerfaces of Al 2 O 3, Ga 2 O 3, In 2 O 3, and related group-III oxide polymorphs: A first-principles study. Phys. Rev. Mater. 2019, 3, 084605 10.1103/PhysRevMaterials.3.084605.

[ref56] Dreyer C. E.; Janotti A.; Van de Walle C. G. Brittle fracture toughnesses of GaN and AlN from first-principles surface-energy calculations. Appl. Phys. Lett. 2015, 106, 212103 10.1063/1.4921855.

[ref57] Adachi K.; Ogi H.; Takeuchi N.; Nakamura N.; Watanabe H.; Ito T.; Ozaki Y. Unusual elasticity of monoclinic β-Ga2O3. J. Appl. Phys. 2018, 124, 085102 10.1063/1.5047017.

[ref58] Griffith A. A.; Taylor G. I. VI. The phenomena of rupture and flow in solids. Philos. Trans. R. Soc. Lond. - Ser. A Contain. Pap. a Math. or Phys. Character 1997, 221 (582–593), 163–198. 10.1098/rsta.1921.0006.

[ref59] Hutchinson J. W.; Suo Z.. In Advances in Applied Mechanics; Hutchinson J. W., Wu T. Y., Eds.; Elsevier, 1991; Vol. 29, pp 63–191.

[ref60] Mattoni A.; Colombo L.; Cleri F. Atomic Scale Origin of Crack Resistance in Brittle Fracture. Phys. Rev. Lett. 2005, 95, 115501 10.1103/PhysRevLett.95.115501. 16197014

