# Supplementary material for: Effective Band Structure and Crack Formation Analysis in Pseudomorphic Epitaxial Growth of (InxGa1–x)2O3 Alloys: A First-Principles Study
Source: ACS Omega. 2024 Mar 20;9(13):15320–7. doi: 10.1021/acsomega.3c10047 (PMC10993274; doi:10.1021/acsomega.3c10047)
Supplement: Supplementary file 1 — ao3c10047_si_001.pdf [file ao3c10047_si_001.pdf]

Supporting Information: Effective Band Structure and Crack Formation Analysis in Pseudomorphic Epitaxial Growth of $(\mathrm{In_xGa_{1-x}})_2\,\mathrm{O_3}$ Alloys: A First Principles Study

Mohamed Abdelilah Fadla,[*,†] Myrta Grüning,[†,¶] and Lorenzo Stella[†,‡]

†*School of Mathematics and Physics, Queen's University Belfast, University Road, Belfast BT7 1NN, United Kingdom*

‡*School of Chemistry and Chemical Engineering, Queen's University Belfast, Stranmillis Road, Belfast BT9 5AG, UK*

¶*European Theoretical Spectroscopy Facility*

E-mail: m.fadla@qub.ac.uk

1. Surface Energies

Slab structures are created using the pymatgen SlabGenerator feature.[1,2] As shown in figure S1 Only non-polar and symmetric terminations were considered for the three orientations: [100], [010], and [001].

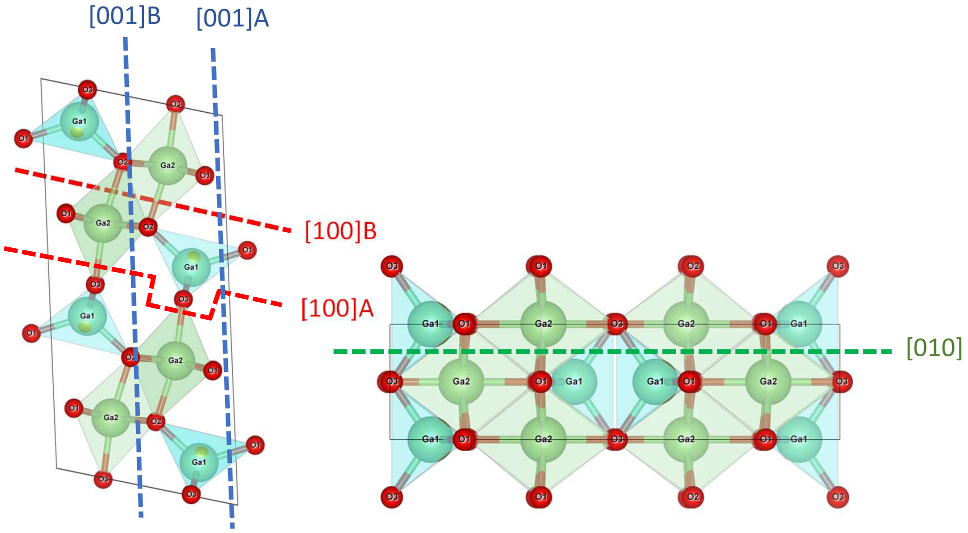

Figure S1: A representation of the 20-atom monoclinic β-Ga_2O_3 conventional unit cell, visualized using VESTA.[3] The figure exhibits (100), (010), and (001) surfaces depicted by red, green, and blue dashed lines respectively, illustrating nonpolar and symmetric terminations. Inequivalent Ga_1 and Ga_2 atoms display tetrahedral (blue) and octahedral (green) coordination, respectively.

The thickness of the slabs is converged with respect to the bulk limit and the convergence of the slab's surface energy (γ) is ensured. This value dictates the surface stability and can be computed using the following equation:[4]

$$\gamma = \frac{1}{2A} \lim_{N \to \infty} \left(E_{\text{slab}} - N E_{\text{bulk}}\right) = \frac{1}{2}\Gamma_b \tag{S1}$$

Where E_{slab} and E_{bulk} are the total energies of slab and bulk systems respectively, N is the number of unit cells in the supercell, A is the supercell cross-section area, and Γ_b is the brittle fracture toughness. In some cases, surface energy does not converge and appears as an oscillation due to differences in computational parameters between bulk and slab, e.g. k-point sampling. To solve this issue, it is highly recommended to use a linear method, where E_{bulk} is determined through a straightforward linear fit. To avoid this numerical error, we have fitted the slab energy.

$$E_{\text{slab}} = 2A\gamma + \text{N}E_{\text{bulk}} \tag{S2}$$

Surface energies are computed using a slab model through linear fitting using Eq. S2. Figure S2 shows the linear fit and the surface energy convergence with respect to the unit cell numbers (thickness).

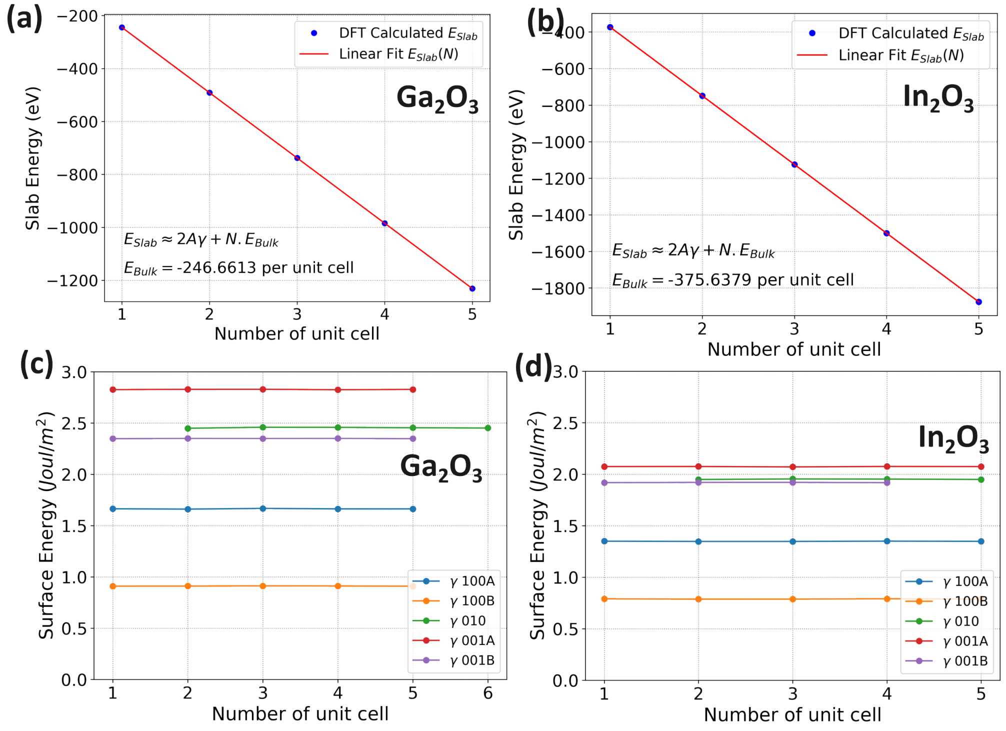

Figure S2: (a,b): A linear model is used to compute the surface energy using linear fitting. Blue circles represent the calculated slab energy (eV) as a function of the unit cell numbers, and the red line shows the fitting line. (c,d): The calculated surface energies (J/m^2) as a function of the number of unit cells show a plateau indicating convergence.

2. Structure

The transformation matrix M, is used to create 2×2×2 supercells (Figure S1.c) for disordered structures, starting from the primitive unit cell (10 atoms; Figure S1.b).

$$M = \begin{pmatrix} 2 & -2 & 0 \\ 2 & 2 & 0 \\ 0 & 0 & 2 \end{pmatrix}$$

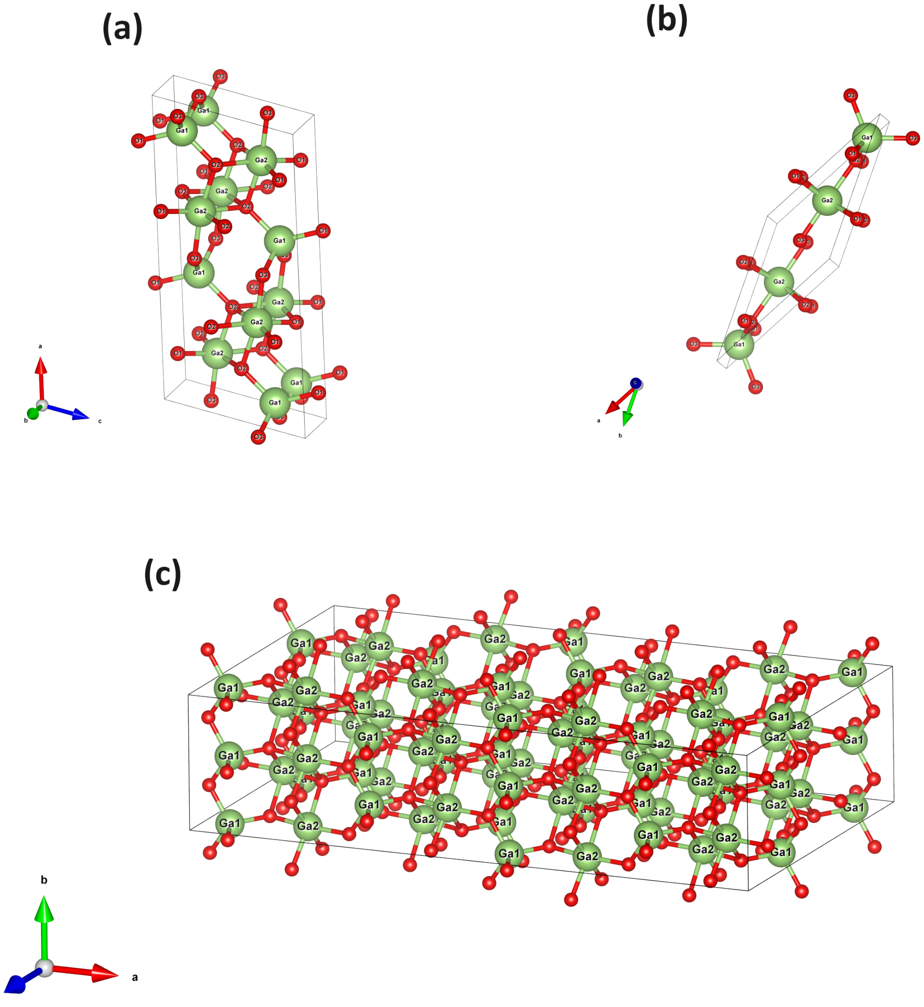

Figure S3: (a) The 20-atom conventional unit cell of monoclinic β-Ga_2O_3. (b) The 10-atom primitive unit cell. (c) The 160-atom 2×2×2 supercell. The inequivalent sites are labelled as Ga_1 and Ga_2, with Ga atoms in green and O atoms in red spheres. The transformation matrix M1 is used to convert the 10-atom primitive unit cell to the 160-atom supercell.

3. Effective band structure

Alloys are constructed using the supercell (SC) approach. For this large system, as the number of bands increases, the band structure is folded into a smaller Brillouin zone, becoming quite complex. Compared to the primitive cell (PC), obtaining meaningful information from the electronic band structure can be challenging, even for small supercells. A band unfolding technique is proposed to compute the effective band structure (EBS) of perturbed systems[5,6]

enabling a feasible comparison between SC and PC.

As the PC and SC are commensurate, we have that $\vec{\mathbf{K}} = \vec{\mathbf{k}}_i - \vec{\mathbf{G}}_i$, with $i = 1, \ldots, N$, where $\vec{\mathbf{K}}$ is a wave vector of the SC, $\vec{\mathbf{k}}_i$ is a wave vector of the PC, and $\vec{\mathbf{G}}_i$ is a reciprocal lattice vector of the SC. Here N is the ratio of the volumes of the PC and SC Brillouin zones. Band unfolding is based on the expression of SC eigenvectors as a linear combination of PC eigenvectors. The spectral weight, defined as

$$P_{\vec{\mathbf{K}}m}\left(\overrightarrow{\mathbf{k}}_i\right) = \sum_n \left|\left\langle \psi_{\vec{\mathbf{K}}m} \mid \psi_{\overrightarrow{\mathbf{k}}_i n} \right\rangle\right|^2 , \tag{S3}$$

determines the probability of an SC eigenstate to have the same Bloch character as a PC eigenstate.[7] From the knowledge of the spectral weight, the spectral function

$$A\left(\vec{\mathbf{k}}_i, E\right) = \sum_m P_{\vec{\mathbf{K}}m}\left(\overrightarrow{\mathbf{k}}_i\right) \delta\left(E - E_{\vec{\mathbf{K}}m}\right) , \tag{S4}$$

is obtained, where $E_{\vec{\mathbf{K}}m}$ is the energy of the SC eigenvector $\psi_{\vec{\mathbf{K}}m}$. The EBS is given by the spectral function.

In Fig. S5 we validate the method by showing the *effective* band structure of pristine β-Ga_2O_3. The spectral function only gives 0 or 1, as expected from a perfectly conserved Bloch character of the eigenvectors.

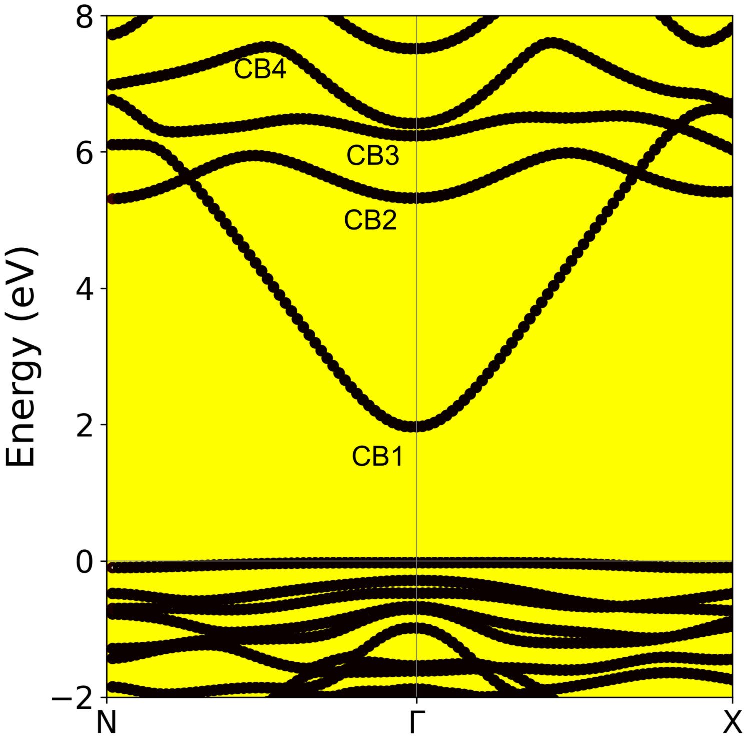

Figure S4: Calculated effective band structure of pristine β-Ga_2O_3 using a 160-atom supercell, plotted for the $N\Gamma X$ Brillouin zone path. The valence band maximum (VBM) is set to zero.

4. Density of states and dielectric function

We computed the density of states and dielectric properties using GGA PBE for a 160-atom supercell. Fig. S5 mainly shows how the band gap decreases as the indium concentration is increased. For all alloys, the peaks around 6 eV are most notably affected by disorder.

The real and imaginary parts of the dielectric function were computed using the independent particle approximation (IPA). Changing the indium fraction x, we observe a redshift of the onset of the imaginary part and an increase of the static dielectric constant (the real part of the dielectric function in the limit of $\omega \to 0$ in the inset of Fig. S6.). As the dielectric function in the IPA reflects the joint density of states, the redshift of the onset of absorption corresponds to the decreasing band gap observed in the DOS. The calculations do not seem to suggest that measuring either the absorption spectrum or the static dielectric function

may provide a measure of the indium fraction x, since the changes are not monotonic (for example we see a clear change when increasing from $x = 0.188$ to $x = 0.25$ but almost no change when increasing from $x = 0.25$ to $x = 0.313$).

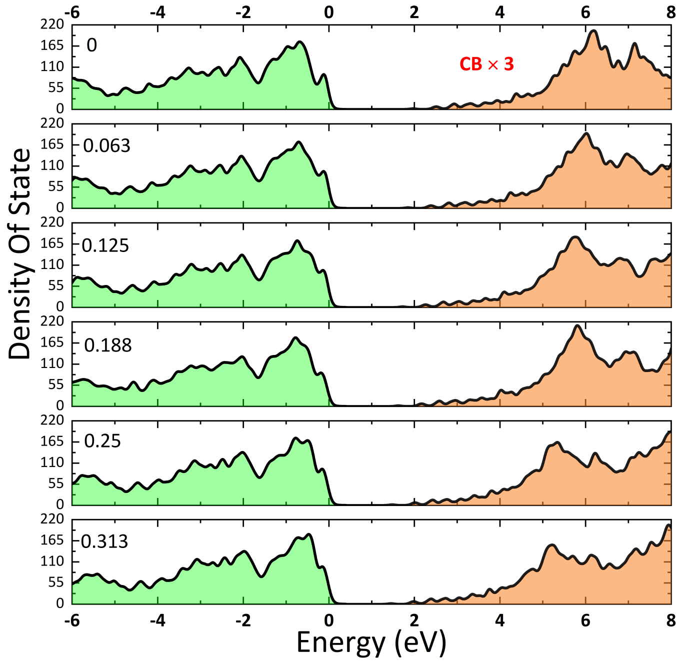

Figure S5: Calculated density of states for β-$(In_xGa_{1-x})_2O_3$ alloys for different indium contents ranging from 0 to 0.31. For clarity, the density of states in the conduction band is scaled by a factor of 3. The zero on the energy axis is placed at the top of the valence band.

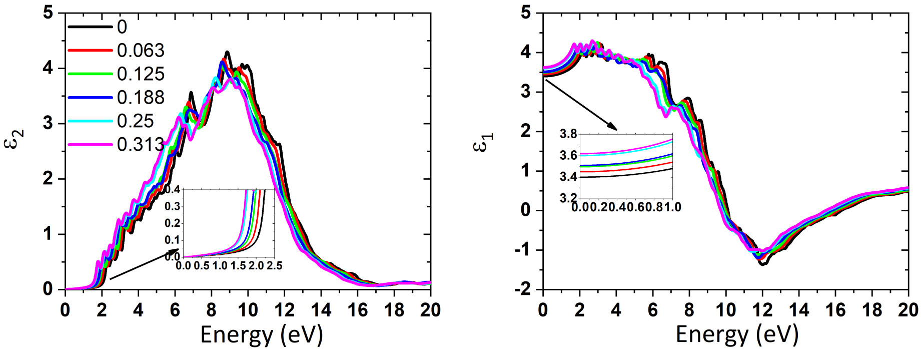

Figure S6: Calculated and real parts of the dielectric function for β-$(In_xGa_{1-x})_2O_3$ alloys, employing the independent particle approximation, within a supercell containing 160 atoms, for various indium contents ranging from 0 to 0.31.

5. Elastic constants and stability

The elastic tensor, C_{ij} is determined by applying Hooke's law from the second-order derivative of total energy versus strain for small cell deformations. In practice, as implemented in the VASPKIT code,[8] small strains, ε_i were applied to the equilibrium lattice constants. Elastic energy, ΔE, is determined from the total energies of the distorted, $E\left(V,\{\varepsilon_i\}\right)$, and undistorted, $E\left(V_0,0\right)$, cells

$$\Delta E\left(V,\{\varepsilon_i\}\right) = E\left(V,\{\varepsilon_i\}\right) - E\left(V_0,0\right) = \frac{V_0}{2}\sum_{i,j=1}^{6} C_{ij}\varepsilon_i\varepsilon_j \ .$$

V and V_0 are the volumes of the distorted and undistorted cells, respectively.

The thirteen independent elastic constants for the monoclinic structures are computed using a 1×2×2 supercell, utilising the SCAN functional. The C_{ij} values are listed for pure β-Ga_2O_3 and other alloys for [0, 0.31] indium content. For comparison, other experimental and theoretical results are presented.

These structures are mechanically stable, as all elastic stability criteria are met.[11] From an experimental point of view, these alloys are known to be stable since have already been synthesized at different concentrations, indicating their stability as powder, films, and ceramics, see Fig. 2 of the main text.

In-plane strain components are computed directly from the mismatch between the lattice constants of the substrate (Ga_2O_3) and films (alloys), and out-of-plane components are computed using a minimization algorithm based on elastic energy.

Table S1: Calculated elastic constant for β-$(In_xGa_{1-x})_2O_3$ alloys for different indium content [0, 0.31]. Pure β-Ga_2O_3 elastic constants are compared with previous experimental and theoretical results.

	β-Ga_2O_3			β-$(In_xGa_{1-x})_2O_3$				
		Ref.[9](Calc.)	Ref.[10](Exp.)	0.063	0.125	0.188	0.25	0.313
C11	251.3	262.1	242.8	247.6	243.4	240.2	237.4	234.6
C22	370.3	374.5	343.8	360.9	348.1	335.1	327.6	314.0
C33	372.6	377.2	347.4	363.1	351.2	337.8	332.7	317.9
C44	57.3	58.9	47.9	157.5	56.9	57.4	52.9	50.4
C55	82.6	77.9	88.6	80.2	75.1	72.0	70.1	67.6
C66	97.0	104.8	104.0	96.1	93.0	87.2	80.5	63.1
C12	129.7	132.2	128.3	125.5	122.1	117.8	113.1	107.5
C13	151.4	140.8	160.0	150.2	148.7	148.6	146.6	146.2
C23	83.5	86.9	70.9	80.1	82.2	77.5	79.5	76.5
C15	-17.2	–21.2	–1.6	-13.2	-12.6	-11.0	-8.4	-7.8
C25	14.9	12.2	0.4	14.3	13.9	13.2	12.9	15.2
C35	10.4	7.5	1.0	11.8	15.3	12.0	13.0	10.1
C46	18.0	19.0	5.6	16.3	15.0	15.2	14.6	14.3

Table S2: The calculated strain components for [100], [010], and [001] growth scenarios for Indium content (x) ranging from 0.063 to 0.313. In-plane components (in bold) are computed based on lattice mismatch, while the out-of-plane components (the remaining four) are computed by minimizing the elastic energy.

In content (x)	Orientation	$\epsilon 1$	$\epsilon 2$	$\epsilon 3$	$\epsilon 4$	$\epsilon 5$	$\epsilon 6$
0.063	100	0.82	**-0.95**	**-0.53**	0.00	0.38	0.00
	010	**-0.38**	0.25	**-0.53**	0.00	-0.03	0.00
	001	**-0.38**	**-0.95**	0.36	0.00	0.05	0.00
0.125	100	1.53	**-1.79**	**-0.98**	0.00	0.79	0.00
	010	**-1.06**	0.60	**-0.98**	0.00	-0.09	0.00
	001	**-1.06**	**-1.79**	0.87	0.00	-0.02	0.00
0.188	100	2.11	**-2.58**	**-1.30**	0.00	1.01	0.00
	010	**-1.75**	0.93	**-1.30**	0.00	-0.22	0.00
	001	**-1.75**	**-2.58**	1.36	0.00	-0.02	0.00
0.25	100	2.53	**-3.31**	**-1.47**	0.00	1.18	0.00
	010	**-2.57**	1.25	**-1.47**	0.00	-0.27	0.00
	010	**-2.57**	**-3.31**	1.92	0.00	-0.06	0.00
0.313	100	3.13	**-3.93**	**-2.04**	0.00	1.55	0.00
	010	**-3.17**	1.60	**-2.04**	0.00	-0.42	0.00
	001	**-3.17**	**-3.93**	2.40	0.00	0.16	0.00

6. k-point Convergence

We utilized a 0.03 $\AA$ separation for the reciprocal k-point grid sampling in our investigation. The PBE generalized gradient approximation was employed for the following convergence study.

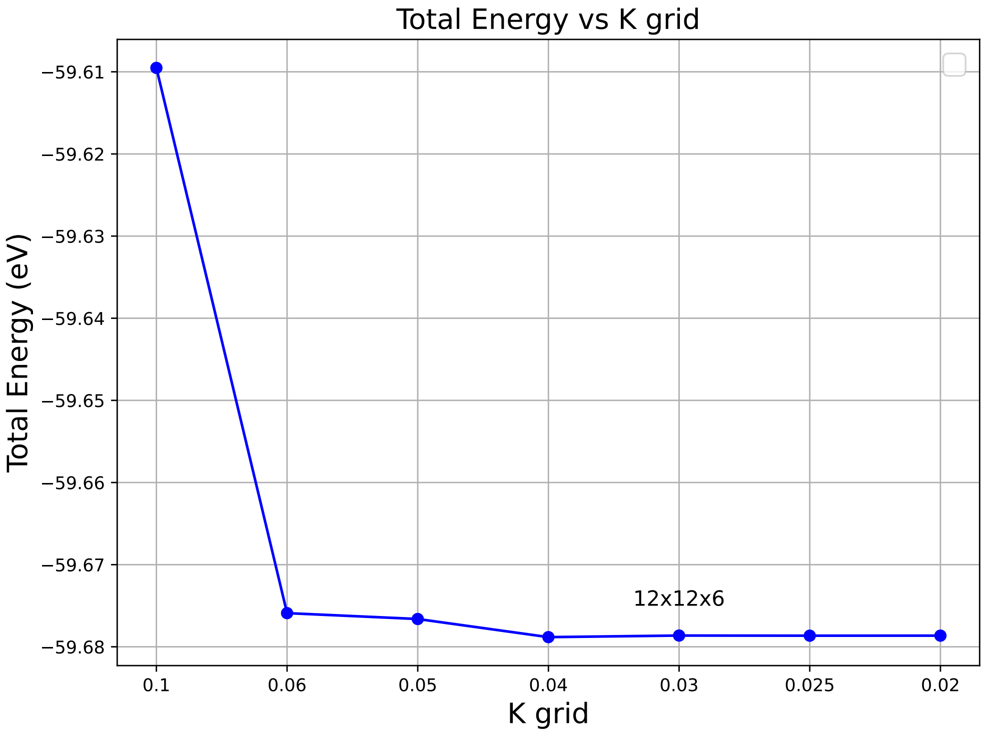

Figure S7: The variation of total energy with respect to different K grid values using the primitive unit cell.

References

(1) Sun, W.; Ceder, G. Efficient creation and convergence of surface slabs. *Surface Science* **2013**, *617*, 53–59.

(2) Ong, S. P.; Richards, W. D.; Jain, A.; Hautier, G.; Kocher, M.; Cholia, S.; Gunter, D.; Chevrier, V. L.; Persson, K. A.; Ceder, G. Python Materials Genomics (pymatgen): A robust, open-source python library for materials analysis. *Computational Materials Science* **2013**, *68*, 314–319.

(3) Momma, K.; Izumi, F. VESTA 3 for three-dimensional visualization of crystal, volumetric and morphology data. *Journal of Applied Crystallography* **2011**, *44*, 1272–1276, Number: 6 Publisher: International Union of Crystallography.

(4) Fiorentini, V.; Methfessel, M. Extracting convergent surface energies from slab calculations. *J. Phys.: Condens. Matter* **1996**, *8*, 6525.

(5) Popescu, V.; Zunger, A. Effective Band Structure of Random Alloys. *Phys. Rev. Lett.* **2010**, *104*, 236403, Publisher: American Physical Society.

(6) Popescu, V.; Zunger, A. Extracting E versus k effective band structure from supercell calculations on alloys and impurities. *Phys. Rev. B* **2012**, *85*, 085201, Publisher: American Physical Society.

(7) Sharma, A.; Singisetti, U. Effective electronic band structure of monoclinic β-(AlxGa1-x)2O3 alloy semiconductor. *AIP Advances* **2023**, *13*, 015101.

(8) Wang, V.; Xu, N.; Liu, J.-C.; Tang, G.; Geng, W.-T. VASPKIT: A user-friendly interface facilitating high-throughput computing and analysis using VASP code. *Computer Physics Communications* **2021**, *267*, 108033.

(9) Mu, S.; Wang, M.; Peelaers, H.; Van De Walle, C. G. First-principles surface energies for monoclinic Ga2O3 and Al2O3 and consequences for cracking of (Al x Ga1- x)2O3. *APL Materials* **2020**, *8*, 091105.

(10) Adachi, K.; Ogi, H.; Takeuchi, N.; Nakamura, N.; Watanabe, H.; Ito, T.; Ozaki, Y. Unusual elasticity of monoclinic β-Ga2O3. *Journal of Applied Physics* **2018**, *124*, 085102.

(11) Mouhat, F.; Coudert, F. m. c.-X. Necessary and sufficient elastic stability conditions in various crystal systems. *Phys. Rev. B* **2014**, *90*, 224104.
